# Deformation Behavior and Constitutive Model of 34CrNi3Mo during Thermo-Mechanical Deformation Process

**DOI:** 10.3390/ma15155220

**Published:** 2022-07-28

**Authors:** Xiang-Dong Jia, Ying Zhou, Yi-Ning Wang

**Affiliations:** College of Mechanical and Electrical Engineering, Nanjing Forestry University, Nanjing 210037, China; zy15161267050@163.com (Y.Z.); 13940572546@139.com (Y.-N.W.)

**Keywords:** flow stress, constitutive model, formability, processing map, 34CrNi3Mo

## Abstract

With higher creep strength and heat resistance, 34CrNi3Mo has been widely used in the production of engine rotors, steam turbine impellers, and turbine blades. To investigate the hot deformation behaviors of 34CrNi3Mo steel, hot compressive tests were conducted on a Gleeble-3500 thermomechanical simulator, under the temperature range of 1073 K–1373 K and strain rate ranges of 0.1 s^−1^–20 s^−1^. The results show that the flow stress of 34CrNi3Mo steel under high temperatures is greatly influenced by the deformation temperature and strain rate, and it is the result of the interaction between strain hardening, dynamic recovery, and recrystallization. Under the same deformation rate, as the deformation temperature increases, the softening effect of dynamic recrystallization and dynamic recovery gradually increases, and the flow stress gradually decreases. Under the same deformation temperature, with the increase of strain rate, the influence of strain hardening on 34CrNi3Mo steel is gradually in power, and the flow stress gradually increases. To predict the flow stress of 34CrNi3Mo steel accurately, a modified Arrhenius-type constitutive model considering the effects of strain, temperature, and strain rate at the same time was made based on the experiment data. On this basis, the evolution law of deformation activation and instability characteristics of 34CrNi3Mo steel were investigated, and the processing map of 34CrNi3Mo steel was established. The formability of 34CrNi3Mo steel under high temperature deformation was revealed, which provided a theoretical foundation of the equation of reasonable hot working process.

## 1. Introduction

With high creep strength and high heat resistance, 34CrNi3Mo steel is a kind of high-strength alloy structural steel widely used in the production of engine rotors, steam turbine impellers, and turbine blades, and these important force transmission parts must be produced by forging to meet its application requirements. In the process of forging, the rheological properties and forming properties of materials at high temperature are the preconditions for working out a reasonable forging process. Therefore, it is important to establish the flow stress model of 34CrNi3Mo at high temperature to reveal its deformation characteristics and forming properties, which is of guiding significance for formulating a reasonable forging process.

A reasonable flow stress model can accurately describe the influence of forming process parameters on the high temperature deformation conditions, such as deformation temperature, deformation amount, and deformation rate, which is also a prerequisite for accurately predicting and analyzing the deformation law of materials in the plastic forming process [1,2]. The macroscopic phenomenological constitutive model based on mechanics of continuous media and thermodynamics describes the mechanical properties of materials by simulating and fitting the macroscopic phenomena of materials in the deformation process [3]. It cannot reveal the characteristics and evolution mechanism of material deformation from an imperceptible and microscopic perspective, however, the modeling and solving process of phenomenological constitutive model is relatively simple, and its application is convenient [4,5,6]. Therefore, the constitutive model based on phenomenological theory is widely used in solving engineering problems. Scholars have modified the original phenomenological model through various technical and theoretical means to improve the prediction accuracy of the model. The Arrhenius-type constitutive model establishes the relationship between flow stress σ, strain rate $\dot{\varepsilon }$, and deformation temperature T, the model parameters can be determined by fitting with experimental data, and the model can be applied to most metal materials [7,8,9]. However, for materials whose rheological behavior is significantly affected by strain, temperature, and strain rate, since the Arrhenius-type model does not consider the influence of temperature, strain rate, and strain on model parameters, the prediction accuracy of the model for this kind of materials is not high. Through research, Li [10] and Safar [11] pointed out that the modified Arrhenius-type constitutive model considering the influence of plastic strain on model parameters could accurately predict the rheological behavior of steel under high temperature deformation. Therefore, the modified Arrhenius-type constitutive model, which can reflect the relationship between the model parameters and temperature, strain rate, and strain, is widely used to evaluate the interaction between the softening effect of dynamic recrystallization and the hardening effect of deformation under thermal deformation [5,12] The Johnson–Cook model is another phenomenological constitutive equation and is used to describe the rheological behavior of materials under high temperature deformation [13]. The traditional Johnson–Cook model combines strain rate hardening with temperature softening effect; however, the model ignores the coupling relationship between strain rate and temperature, resulting in low prediction accuracy [14]. To overcome this shortcoming, scholars established a modified Johnson–Cook model coupled with strain rate and deformation temperature [15]. With the modified Johnson–Cook model, the flow stress of aluminum alloy [16], magnesium alloy [17], and high strength steel [18] is accurately predicted under the condition of high temperature deformation. In addition, the application of an artificial neural network model based on experimental data and phenomena in the prediction of material flow stress is also gradually expanding [19]. Through comparative studies, scholars, such as Wang [20], Ashtiani [21] and Quan [22], have found that the artificial neural network (ANN) model could accurately predict the deformation behavior of metal materials under the thermal deformation condition.

In the process of hot deformation, in addition to determining the rheological behavior of materials and establishing the constitutive mode, the instability behavior of materials under high temperature is also an important criterion for reasonable process parameters [23,24]. In the instability theory of high temperature deformation, the processing map is the main theoretical basis for predicting the instability characteristics of materials. Due to the limitation of the atomic model, the processing map (dynamic material model) based on Prasad dynamic material model [25] and Murty instability criterion [26] has been widely used in engineering. Based on the dynamic material model, scholars studied the instability characteristics of various metal materials such as aluminum alloy [27], magnesium alloy [28], titanium alloy [29], superalloy [30], and stainless steel [31] during hot deformation, and established the processing maps of various metal materials, which can be used to guide the selection and determination of reasonable thermal processing parameters.

Therefore, in order to reveal the rheological stress and instability characteristics of 34CrNi3Mo steel during hot deformation, the rheological characteristics and the processing map of 34CrNi3Mo steel under different deformation conditions were revealed in the article based on physical experiment and theoretical models. The research results will provide a theoretical and experimental basis for the determination of reasonable thermal processing parameters of 34CrNi3Mo steel.

## 2. Experiment

### 2.1. Experiment Method

**Figure 1:**
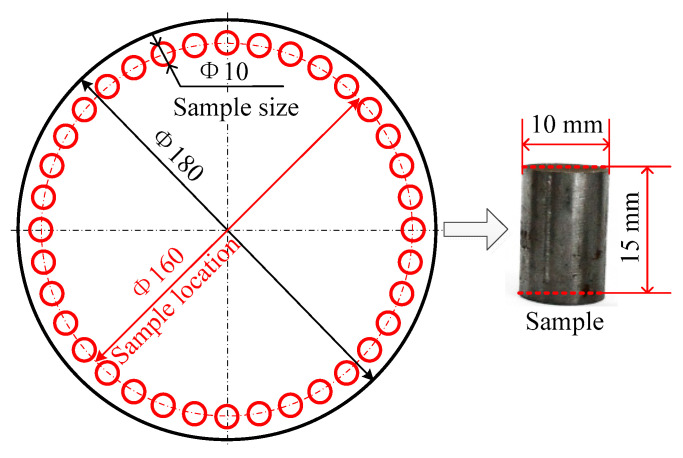
Size and position of test specimens.

**Figure 2:**
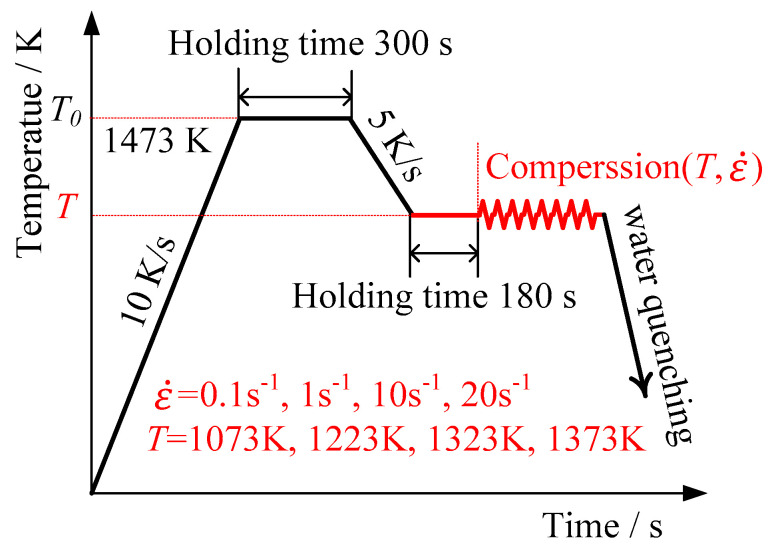
Thermal simulation process curve.

In this paper, 34CrNi3Mo hot-rolled bar with a diameter of 180 mm was taken as the research object. In order to eliminate the difference in material properties caused by the difference in internal microstructure, a compression sample with a diameter of 10 mm and a height of 15 mm were cut at the position on the outer surface of the bar, as shown in Figure 1, under the deformation conditions of four different temperatures (1073 K, 1223 K, 1323 K, 1373 K) and four different strain rates (0.1 s^−1^, 1 s^−1^, 10 s^−1^, 20 s^−1^). In the experiment process, the amount of total strain of the compressed sample was set as 0.65, and the isothermal compression tests were carried out on 34CrNi3Mo cylindrical specimens with the help of Gleebel-3500 thermal simulation test machine, as shown in Figure 2.

In forging process, the 34CrNi3Mo workpiece is usually heated to the initial forging temperature (1473 K). However, due to the heat exchange among the workpiece, the mold, and the environment, the temperature of the workpiece gradually decreases. In order to simulate the effect of material temperature change on the properties during forging process, the samples were heated to the temperature T_0_ = 1473 K, then cooled to the deformation temperature and kept there before deformation for 180 s to make the sample temperature uniform.

### 2.2. Results and Analysis

**Figure 3:**
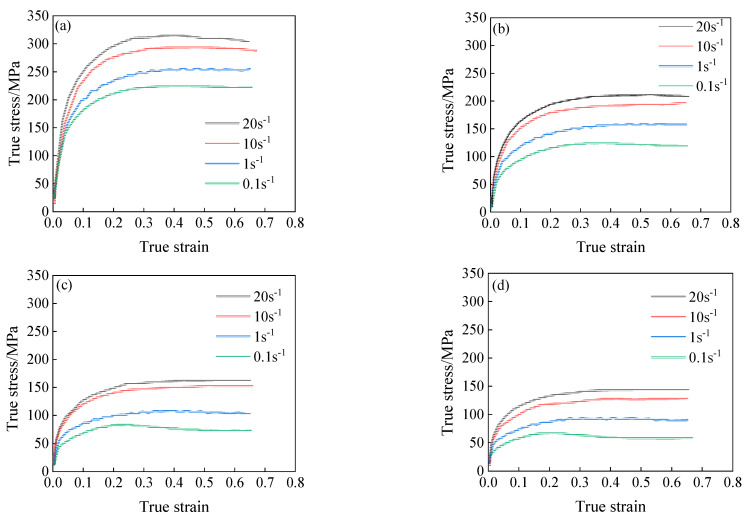
True stress-strain curves of 34CrNi3Mo steel, (**a**) *T* = 1073 K, (**b**) *T* = 1223 K, (**c**) *T* = 1323 K, (**d**) *T* = 1373K.

The true stress-strain curves of 34CrNi3Mo steel at different deformation conditions were obtained through isothermal compression tests, as shown in Figure 3. It can be seen from the test results that the flow stress of 34CrNi3Mo steel under high temperature deformation is the result of the interaction of strain hardening, dynamic recovery, and recrystallization. The flow stress of 34CrNi3Mo steel exhibits obvious dynamic recrystallization under high temperature deformation, especially under high temperature and low strain rate deformation. In the initial stage of deformation, the flow stress increases gradually with the increase of the strain; when the stress reaches a peak value, the flow stress decreases with the increase of the strain.

Under different compression temperature conditions, the flow stress of 34CrNi3Mo steel is greatly affected by the deformation temperature at the same strain rate. With the increase of deformation temperature, the dislocation activity and the kinetic energy of atoms increases at the same time. These microscopic changes will make dislocation slip more easily. Therefore, with the increase of temperature, the softening effect of dynamic recrystallization and dynamic recovery of 34CrNi3Mo steel gradually increases, and the flow stress gradually decreases.

At the same deformation temperature, the dislocation proliferation rate caused by strain hardening increases gradually with the increase of strain rate, while the rate of dislocation disappearance caused by dynamic recovery gradually decreases. Although dynamic recrystallization is easier to nucleate under high dislocation density and high temperature, dynamic recrystallization is not enough to eliminate strain hardening caused by increased dislocation density because of too fast strain rate to short deformation time. Therefore, under the condition of high temperature deformation, with the increase of strain rate, the influence of strain hardening on 34CrNi3Mo steel gradually dominates, and the flow stress gradually increases.

## 3. Constitutive Model

### 3.1. Arrhenius-Type Constitutive Model

It can be seen from the thermal compression test results that the flow stress of 34CrNi3Mo steel is greatly affected by strain, strain rate, and temperature under thermal deformation conditions. In order to accurately describe the flow stress under thermal deformation conditions, the relationship between stress and strain, temperature, and strain rate must be established. The Arrhenius-type constitutive model can characterize the relationship between the flow stress *σ*, strain rate $\dot{\varepsilon }$, and deformation temperature *T* when materials are deformed at high temperature,

(1) $$\dot{\varepsilon }=Af\left(\sigma \right)\cdot \exp\left[-Q/\left(RT\right)\right]\;$$

where *A* is the material parameter, *R* = 8.31J/mol·K, and f(σ) is the stress function.

Under the condition of low stress levels, namely, α*σ* < 0.8, $f\left(\sigma \right)=\sigma^{n_{1}}$. Where α is the stress level parameter (MPa^−1^). Then, substituted into Equation (1), the relationship between flow stress *σ* and strain rate $\dot{\varepsilon }$ can be approximately shown as,

(2) $$\dot{\varepsilon }=A\sigma^{n_{1}}\exp\left[-Q/\left(RT\right)\right]$$

where *n*_1_ is the material parameter.

Under the condition of high stress levels, namely, *ασ* > 1.2, f(σ)=exp(βσ), the flow stress *σ* and strain rate $\dot{\varepsilon }$ can be expressed as the Equation as follows.

(3) $$\dot{\varepsilon }=A_{2}\exp\left[\beta \sigma \right]\cdot \exp\left[-Q/\left(RT\right)\right]$$

where *β* is the constant relevant to the materials, and *β* = *α*·*n*_1_.

Under all stress levels conditions, $f\left(\sigma \right)={\left[\sin h\left(\alpha \sigma \right)\right]}^{n}$, *n* is the material parameter. The Arrhenius-type hyperbolic sine form of constitutive equation containing deformation activation energy *Q* and deformation temperature *T* can be obtained,

(4) $$\dot{\varepsilon }=A{\left[\sinh\left(\alpha \sigma \right)\right]}^{n}\cdot \exp\left[-Q/\left(RT\right)\right]\;$$

By taking logarithms of both sides of Equations (2) and (3), we can see that,

(5) $$ln\sigma =\frac{1}{n_{1}}ln\dot{\varepsilon }+\frac{1}{n_{1}}\left(\frac{Q}{RT}-lnA\right)$$

(6) $$\sigma =\frac{1}{\beta }ln\dot{\varepsilon }+\frac{1}{\beta }\left(\frac{Q}{RT}-lnA\right)$$

From Equations (5) and (6), it can be seen that when the temperature is fixed, the $ln\dot{\varepsilon }$ satisfies the linear equation with σ and lnσ, respectively, namely both $ln\dot{\varepsilon }$-*σ* and $ln\dot{\varepsilon }$-lnσ show a linear relationship. The slope of the linear equation shown in Equations (5) and (6) is the stress index *n*_1_ and the material constant *β*. Therefore, under certain strain conditions, the test results under the same deformation temperature and different strain rates were linearly fitted according to Equation (5) and Equation (6), respectively, then the stress index *n*_1_ and material constant *β* under different stress levels can be obtained.

**Figure 4:**
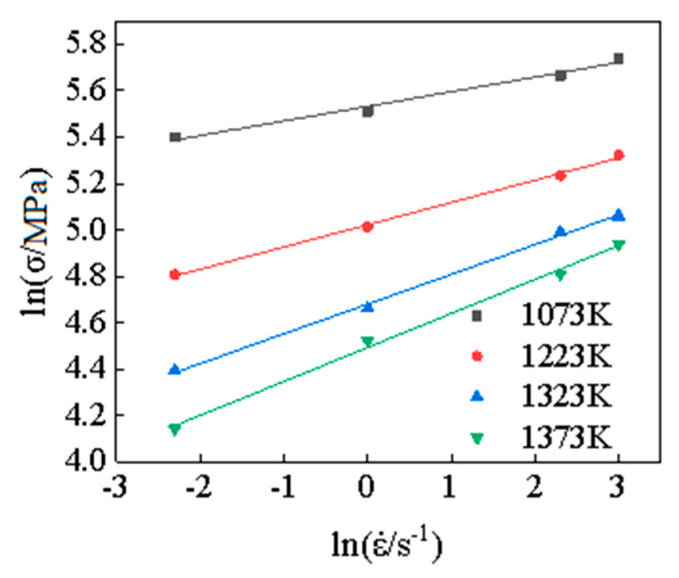
The fitting curves for $\ln\sigma -\ln\dot{\varepsilon }$.

**Figure 5:**
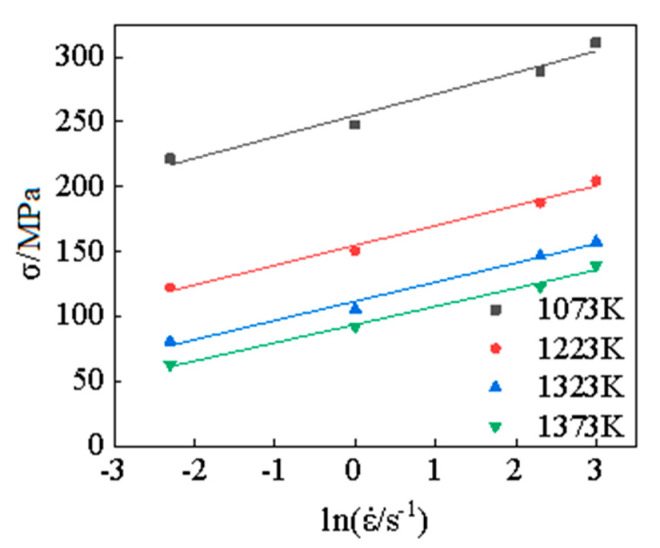
The fitting curves for $\sigma -\ln\dot{\varepsilon }$.

The experimental results of plastic strain *ε* = 0.3 were selected as the research object, and the experimental results were linearly fitted according to Equation (5) and Equation (6), respectively. The linear fitting results of $ln\sigma -ln\dot{\varepsilon }$ and σ-$ln\dot{\varepsilon }$ are obtained under different deformation conditions, as shown in Figure 4 and Figure 5, then the values of the material parameter *n*_1_, *β*, and stress level parameter *α* can be obtained under different conditions, as shown in Table 1.

Since Equation (5) is obtained under the condition of a low stress level, Equation (6) is obtained under the condition of a high stress level. Therefore, we take the average value of the four lines as the value of *n*_1_ in the research process of this paper, the value *n*_1_ = 10.231 can be obtained through calculation. In a similar way, taking the average of the four lines as the value of *β,* thus *β* = 0.0662. According to the relationship between the material parameters *n*_1_ and *β*, *β* = *α*·*n*_1_, the stress level parameter *α*=0.0064MPa^−1^ can be obtained.

The deformation activation energy *Q* can be expressed as,

(7) $$Q=R{\left\{\frac{\partial \ln\dot{\varepsilon }}{\partial \ln\left[\sinh\left(\alpha \sigma \right)\right]}\right\}}_{T}{\left\{\frac{\partial ln\left[sinh\left(\alpha \sigma \right)\;\right]}{\partial \left(1/T\right)}\right\}}_{\dot{\varepsilon }}=RnK\;\;$$

It can be seen from Equation (7) that at a certain deformation temperature *T*, the slope of the curve $\ln\dot{\varepsilon }-\ln\left[\sin h\left(\alpha \sigma \right)\right]$ is the material parameter *n*. When the strain rate $\dot{\varepsilon }$ is fixed, the slope of the curve ln[sinh(ασ)]−ln(1/T) is material parameter *K*, and *K* can be expressed as K=Q/nR.

**Figure 6:**
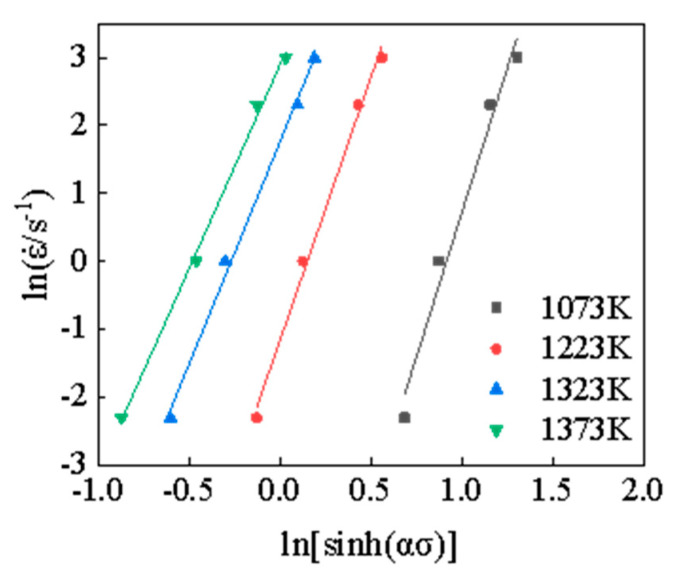
The relation curve of $\ln\dot{\varepsilon }-\ln\left[\sin h\left(\alpha \sigma \right)\right]$.

**Figure 7:**
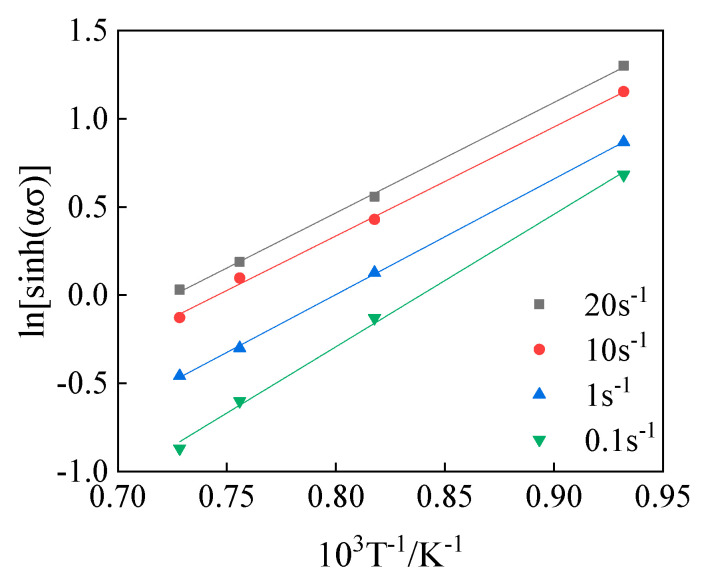
The relation curve of ln[sinh(ασ)]−ln(1/T).

Combining the obtained value *α* and the experimental results obtained under different deformation conditions, the linear fitting results of $ln\dot{\varepsilon }-ln\sinh\left(\alpha \sigma \right)$ and ln[sinh(ασ)]− ln(1/T) can be plotted, as shown in Figure 6 and Figure 7. Thus, the values of *n* under different temperatures can be calculated according to Figure 6 and Figure 7, which is shown in Table 2, and the deformation activation energy *Q* at different strain rates is shown in Table 3.

When the materials produce plastic deformation under high temperature conditions, the relationship between strain rate, deformation activation energy, and deformation temperature can be expressed by the Zener–Hollomon parameter, namely, *Z* parameter representing the strain rate factor of temperature compensation.

(8) $$Z=\dot{\varepsilon }\exp\left[-Q/\left(RT\right)\right]=A{\left[\sinh\left(\alpha \sigma \right)\right]}^{n\;}\;$$

Taking the logarithm of both sides of Equation (8), the Equation (8) will be changed as,

(9) lnZ=n[sinh(ασ)]+lnA

**Figure 8:**
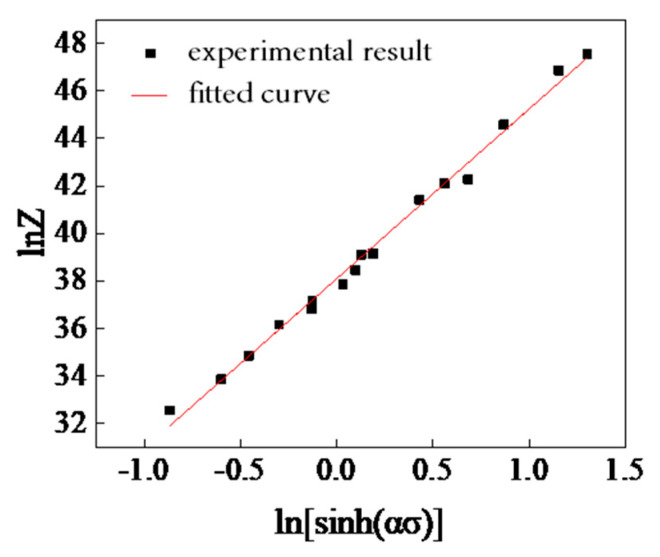
The relation curve of lnZ−ln[sinh(ασ)]..

According to Equation (9), ln*A* is the intercept of the linear curve of lnZ−ln[sinh(ασ)]. By substituting the value of *Q* into the Equation (8), the *Z* parameters under different deformation conditions can be obtained. By linear fitting the relationship between lnZ and ln[sinh(ασ)] under different deformation conditions, as shown in Figure 8, the material parameter *A* can be obtained, *A* = 3.51 × 10^16^.

**Figure 9:**
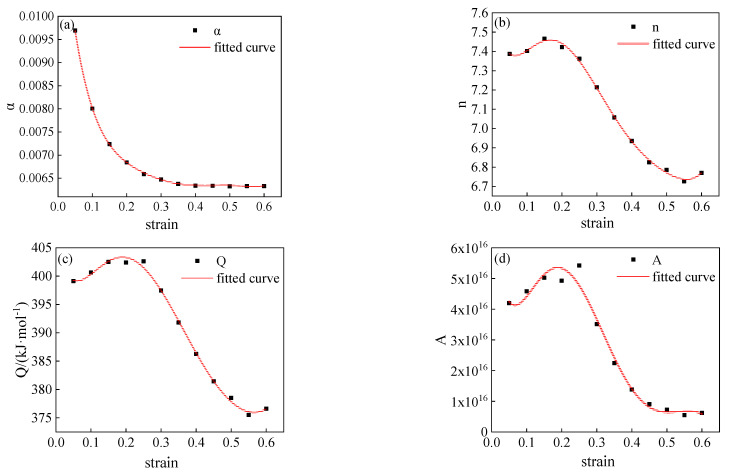
The fitting relationship curve between strain and material parameters: (**a**) α-ε; (**b**) n-ε; (**c**) Q-ε; (**d**) A-ε.

According to the experimental results of 34CrNi3Mo steel, it can be seen that the flow stress curve of 34CrNi3Mo steel is not only related to the deformation temperature and strain rate but is also affected by the plastic strain. Therefore, all the parameters in the Arrhenius-type model will be affected by plastic strain. With the same method as above, a strain value is selected every 0.05 within the strain range of 0.1–0.6, and the values of model parameters under different strain conditions can be calculated, as shown in Table 4. In this paper, the sixth-degree polynomial was used to fit model parameters under different deformation conditions, the fitting curve is shown in Figure 9, and the functional relationship between model parameters and strain obtained through fitting is shown in Equation (10). The coefficients of each material parameter model in Equation (10) are shown in Table 5.

As shown in Table 4 and Figure 9, in the initial stage of plastic deformation, the stress level parameter *α* in the Arrhenius-type constitutive model decreases rapidly with the increase of deformation. When the strain is greater than 0.3, the stress level parameter *α* tends to be stable with the increase of deformation. When the strain is less than 0.2, the material parameter *n*, the deformation activation energy *Q*, and the parameter *A* all increase with the increase of deformation. However, the strain is greater than 0.2 and less than 0.5, the material parameters *n*, *A*, and the deformation activation energy *Q* gradually decrease with the increase of deformation. This change rule will not exist when the strain is greater than 0.5, the three parameters increase gradually with the increase of strain.

(10) $$\left\{ \begin{array}{l} \alpha \left(\varepsilon \right)=B_{0}+B_{1}\varepsilon +B_{2}\varepsilon^{2}+B_{3}\varepsilon^{3}+B_{4}\varepsilon^{4}+B_{5}\varepsilon^{5}+B_{6}\varepsilon^{6}\\ n\left(\varepsilon \right)=C_{0}+C_{1}\varepsilon +C_{2}\varepsilon^{2}+C_{3}\varepsilon^{3}+C_{4}\varepsilon^{4}+C_{5}\varepsilon^{5}+C_{6}\varepsilon^{6}\\ Q\left(\varepsilon \right)=D_{0}+D_{1}\varepsilon +D_{2}\varepsilon^{2}+D_{3}\varepsilon^{3}+D_{4}\varepsilon^{4}+D_{5}\varepsilon^{5}+D_{6}\varepsilon^{6}\\ A\left(\varepsilon \right)=E_{0}+E_{1}\varepsilon +E_{2}\varepsilon^{2}+E_{3}\varepsilon^{3}+E_{4}\varepsilon^{4}+E_{5}\varepsilon^{5}+E_{6}\varepsilon^{6} \end{array}\right.$$

According to Equation (4), the flow stress can be expressed as,

(11) $$\sigma =\frac{1}{\alpha \left(\varepsilon \right)}\sinh^{-1}{\left\{\frac{\dot{\varepsilon }\exp\left[\frac{Q\left(\varepsilon \right)}{RT}\right]}{A\left(\varepsilon \right)}\right\}}^{\frac{1}{n\left(\varepsilon \right)}}$$

**Figure 10:**
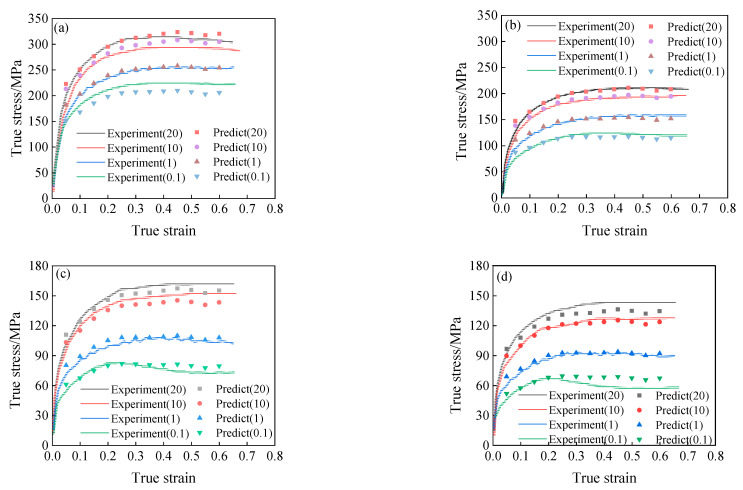
The predicted results of flow stress, (**a**) T = 1073 K; (**b**) T = 1223 K; (**c**) T = 1323 K; (**d**) T = 1373 K.

By substituting the stress level parameters *α*(*ε*), the material parameters *n*(*ε*), *A*(*ε*), and the deformation activation energy *Q*(*ε*) into Equation (11), the prediction results of flow stress under the different conditions can be obtained, as shown in Figure 10.

By comparing the predicted flow stress curve shown in Figure 10 with the experimental results, the Arrhenius-type constitutive model considering the effect of strain can accurately predict the flow stress of 34CrNi3Mo steel when the deformation temperature is lower (less than 1223 K). However, with the increase of deformation temperature, the interaction between dynamic recrystallization and strain rate hardening is gradually obvious, under the condition of low strain rate, the softening phenomenon of the Arrhenius-type constitutive model considering strain only is not obvious, and the strain rate hardening effect cannot be reflected under the higher strain rate. Therefore, there is a large error between model prediction results and experimental results at high temperature.

### 3.2. Modified Arrhenius-Type Constitutive Model

It can be seen from the above research that, in order to accurately predict the flow stress of 34CrNi3Mo steel under high temperature deformation with the help of the Arrhenius-type constitutive model, the interaction between strain, deformation temperature, and strain rate on model parameters must be considered simultaneously. Therefore, the modified Arrhenius-type model can be rewritten as [18],

(12) $$\dot{\varepsilon }=A\left(\varepsilon ,\;\dot{\varepsilon },T\right){\left\{\sin h\left[\alpha \left(\varepsilon ,T\right)\sigma \right]\right\}}^{n\left(\varepsilon ,T\right)}\exp\left[-Q\left(\varepsilon ,\;\dot{\varepsilon },T\right)/RT\right]$$

where the material parameter *n* and the stress level parameter *α* are the functions of strain and deformation temperature, namely, n(ε,T), α(ε,T), the deformation activation energy *Q* and the material parameter *A* are the functions of strain, strain rate, and deformation temperature, namely, $A\left(\varepsilon ,\;\dot{\varepsilon },T\right)$, $Q\left(\varepsilon ,\;\dot{\varepsilon },T\right)$.

**Figure 11:**
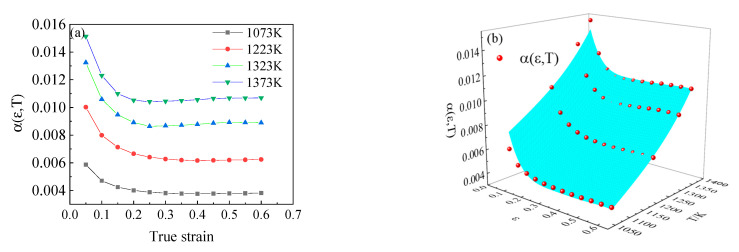
The value of α(ε,T) under different deformation conditions: (**a**) the experimental results, (**b**) the fitting results.

The value of stress level parameter *α* under different deformation temperatures and strains are shown in Figure 11a. It can be seen from the figure that stress level parameters are greatly affected by deformation temperature. After undergoing large plastic deformation, strain has little influence on stress level parameters. Through nonlinear surface fitting, as shown in Figure 11b, the function of stress level parameter *α* on deformation temperature *T* and strain ε can be expressed as follows,

(13) $$\alpha \left(\varepsilon ,T\right)=0.03419-0.01172\varepsilon -0.000065T+0.02498\varepsilon^{2}+3.7\times 10^{-8}T^{2}-6.9\times 10^{-6}\varepsilon T$$

According to the Equation of the correlation coefficient, the correlation coefficient *R*^2^ of the fitted surface is 0.951. This proves the accuracy of fitting formula.

Taking the logarithm of both sides of Equation (12), we can see that,

(14) $$\ln\dot{\varepsilon }=n\left(\varepsilon ,\;T\right)\ln\left\{\sin h\left[\alpha \left(\varepsilon ,T\right)\sigma \right]\right\}+\left[\frac{-Q\left(\varepsilon ,\;\dot{\varepsilon },T\right)}{RT}+\mathrm{lnA}\left(\varepsilon ,\;\dot{\varepsilon },T\right)\right]$$

**Figure 12:**
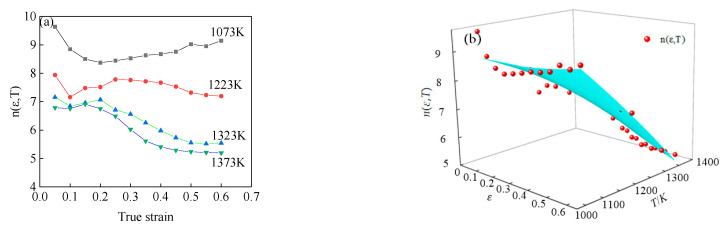
The value of *n*(*ε*,*T*) under different deformation conditions: (**a**) the experimental results, (**b**) the fitting results.

According to Equation (14), *n*(*ε,T*) is the slope of curves $\ln\dot{\varepsilon }$- under different strains and deformation temperatures. Therefore, according to the flow stress curve of 34CrNi3Mo steel, the curves of *n*(*ε,T*) under different strain and temperature conditions can be drawn, as shown in Figure 12a. It can be seen from Figure 12a that the value of *n*(*ε,T*) is greatly affected by strain and temperature. Under the same strain condition, the value of *n*(*ε,T*) decreases obviously with the increase of temperature. At the same deformation temperature, when the deformation temperature is greater than 1223 K, the value of *n*(*ε,T*) decreases with the increase of strain.

Similary, the function of material parameter *n* on deformation temperature *T* and strain *ε* was obtained by 3D surface fitting, as shown in Figure 12b, and the expression of fitting function is shown in Equation (15), with the correlation coefficient *R*^2^ = 0.951.

(15) $$n\left(\varepsilon ,T\right)=7.66322+14.48418\varepsilon +0.00582T+1.68993\varepsilon^{2}-0.00000446633T^{2}-0.01402\varepsilon T$$

Under the certain strain and deformation rate, Equation (14) can be rewritten as,

(16) $$\frac{Q\left(\varepsilon ,\;\dot{\varepsilon },T\right)}{Rn\left(\varepsilon ,\;T\right)}\frac{1}{T}=\frac{\mathrm{lnA}\left(\varepsilon ,\;\dot{\varepsilon },T\right)-ln\varepsilon }{n\left(\varepsilon ,\;T\right)}+\ln\left\{\sin h\left[\alpha \left(\varepsilon ,T\right)\sigma \right]\right\}$$

According to Equation (16), it can be seen that the deformation activation energy *Q* is the slope of the curve of ln{sinh[α(ε)σ]} − 1/*T.*

(17) $$Q\left(\varepsilon ,\;\dot{\varepsilon },T\right)=1000Rn\left(\varepsilon ,\;T\right){\left\{\frac{\partial \mathrm{lnA}\left(\varepsilon ,\;\dot{\varepsilon },T\right)-ln\varepsilon }{\partial n\left(\varepsilon ,\;T\right)}\right\}}_{\varepsilon ,\dot{\varepsilon }}=1000Rn\left(\varepsilon ,\;T\right)K\left(\varepsilon ,\;\dot{\varepsilon }\right)$$

According to Equation (17), the deformation activation energy $Q\left(\varepsilon ,\;\dot{\varepsilon },T\right)$ consists of two functions, n(ε, T) and $K\left(\varepsilon ,\;\dot{\varepsilon }\right)$. The function of n(ε, T) can be obtained according to Equation (15), and the $K\left(\varepsilon ,\;\dot{\varepsilon }\right)$ is a function of strain and strain rate.

**Figure 13:**
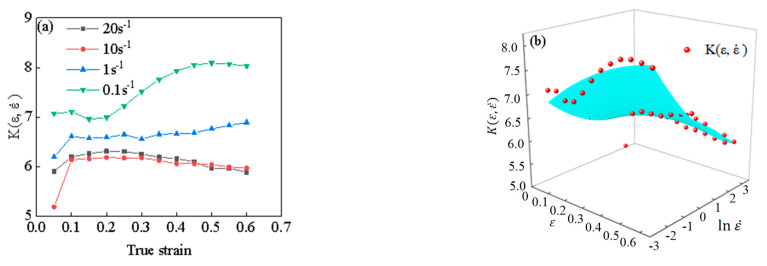
The value of *K*(*ε*, $\dot{\varepsilon }$) under different deformation conditions: (**a**) the calculation results, (**b**) the fitting results.

According to the experimental results, the value of $K\left(\varepsilon ,\;\dot{\varepsilon }\right)$ under different strains and strain rates can be obtained through calculation, the calculation results are shown in Figure 13a. It can be seen from the figure that the strain and strain rate have great influence on the value of $K\left(\varepsilon ,\;\dot{\varepsilon }\right)$. Under the same strain condition, the value of $K\left(\varepsilon ,\;\dot{\varepsilon }\right)$ decreases gradually with the increase of strain rate. When the strain rate is small ($\dot{\varepsilon }$ ≤ 1 s^−1^), the value of $K\left(\varepsilon ,\;\dot{\varepsilon }\right)$ decreases with the increase of deformation (ε > 0.1). However, when the strain rate is large ($\dot{\varepsilon }$ ≥ 10 s^−1^), the value of $K\left(\varepsilon ,\;\dot{\varepsilon }\right)$ increases gradually with the increase of deformation (ε > 0.1). The functional expression of the function of $K\left(\varepsilon ,\;\dot{\varepsilon }\right)$ can be obtained through 3D surface fitting, as shown in Figure 13b. The fitting function is shown in Equation (18), and the correlation coefficient *R*^2^ of the fitting results is 0.937.

(18) $$K\left(\varepsilon ,\;\dot{\varepsilon }\right)=5.99546+3.06765\varepsilon -0.16104\ln\;\dot{\varepsilon }-2.92598\varepsilon^{2}+0.04702{\left(\ln\;\dot{\varepsilon }\right)}^{2}-0.48355\varepsilon \left(\ln\;\dot{\varepsilon }\right)$$

By substituting Equation (18) and Equation (15) into Equation (17), the functional expression of the deformation activation energy $Q\left(\varepsilon ,\;\dot{\varepsilon },T\right)$ considering the effects of strain, strain rate, and deformation temperature can be obtained.

**Figure 14:**
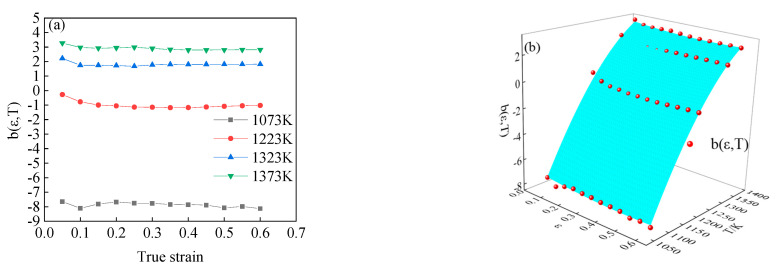
The value of *b*(*ε,T*) under different deformation conditions: (**a**) the calculation results, (**b**) the fitting results.

According to Equation (14), assuming the function value of b(ε,T) is equal to the function value of $\mathrm{lnA}\left(\varepsilon ,\;\dot{\varepsilon },T\right)-Q\left(\varepsilon ,\;\dot{\varepsilon },T\right)/RT$, thus, the value of b(ε,T) is the intercept of the curve $\ln\dot{\varepsilon }$-ln{sinh[α(ε,T)σ]} under the different deformation temperatures and strains. Therefore, the value of b(ε,T) could be calculated according to the experimental results, as shown in Figure 14a. It can be seen from the figure that under the same strain condition, the value of b(ε,T) increases gradually with the increase of deformation temperature. However, at the same deformation temperature, the variation of b(ε,T) value is just opposite to the previous rule, the value of b(ε,T) decreases with the increase of strain. The function of b(ε,T) can be obtained through 3D surface fitting, as shown in Figure 14b. The expression of fitting function b(ε,T) is shown in Equation (19), with the correlation coefficient *R*^2^ = 0.999.

(19) $$b\left(\varepsilon ,T\right)=-141.54581-2.60296\varepsilon +0.19471T+2.3216\varepsilon^{2}-0.0007T^{2}+0.00045\varepsilon T$$

By substituting Equations (19) and (17) into the equation of $b\left(\varepsilon ,T\right)=\mathrm{lnA}\left(\varepsilon ,\;\dot{\varepsilon },T\right)-Q\left(\varepsilon ,\;\dot{\varepsilon },T\right)/RT$, the expression of $\mathrm{lnA}\left(\varepsilon ,\;\dot{\varepsilon },T\right)$ can be obtained,

(20) $$\mathrm{lnA}\left(\varepsilon ,\;\dot{\varepsilon },T\right)=b\left(\varepsilon ,T\right)+\frac{1000n\left(\varepsilon ,\;T\right)K\left(\varepsilon ,\;\dot{\varepsilon }\right)}{T}\;\;$$

Using a similar method as in Equation (4), the rheological prediction model considering the interaction between the stress with plastic strain, temperature, and strain rate can be obtained by reordering and transforming the Equation (12), the prediction model is shown in Equation (21), where the parameters of the model could be calculated through Equations (13) and (17)–(20).

(21) $$\sigma =\frac{1}{\alpha \left(\varepsilon ,T\right)}\sinh^{-1}{\left\{\frac{\dot{\varepsilon }\exp\left[\frac{Q\left(\varepsilon ,\dot{\varepsilon },T\right)}{RT}\right]}{A\left(\varepsilon ,\dot{\varepsilon },T\right)}\right\}}^{\frac{1}{n\left(\varepsilon ,T\right)}}\;$$

**Figure 15:**
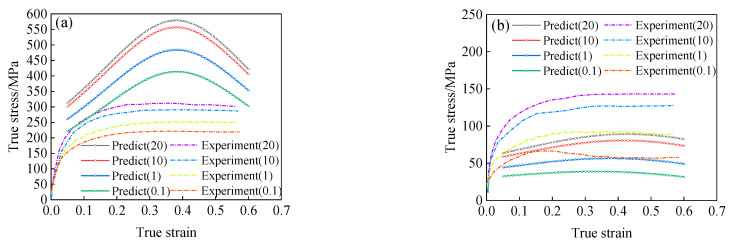
The predicted results based on the Equation (21): (**a**) *T* = 1073 K; (**b**) *T* = 1373 K.

According to the constitutive model of Equation (21), the predicted results of the flow stress of 34CrNi3Mo steel under the high temperature condition are shown in Figure 15. Comparing the predicted results according to Equation (21) with the experimental results, as shown in Figure 15, the predicted results have a large error with the experimental results. For exploring the causes of such errors, the effects of material parameters n(ε,T), $A\left(\varepsilon ,\dot{\varepsilon },T\right)$, stress level parameter α(ε,T), and deformation activation energy $Q\left(\varepsilon ,\dot{\varepsilon },T\right)$ on the constructive model (as shown in Equation (21)) were investigated. Through comparison research, it was found that stress level parameters α(ε,T) were the main reasons for the large error.

**Figure 16:**
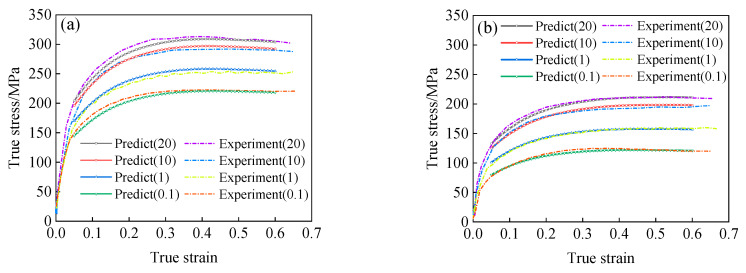

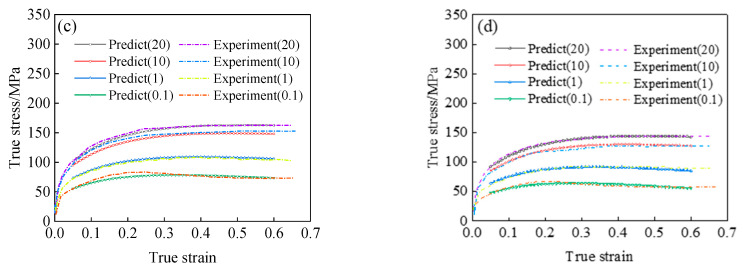
The predicted results of the modified Arrhenius-type model: (**a**) T = 1073 K, (**b**) T = 1223 K, (**c**) T = 1323 K, (**d**) T = 1373 K.

When the stress level parameter is only related to strain, and other parameters function of Equation (21) remain the same, that is to say, in the calculation of the flow stress by using Equation (21), just replace the function of level parameter α(ε,T) with α(ε), the expression of the α(ε) satisfies Equation (10). Through the transformation mentioned above, the predicted results of flow stress are shown in Figure 16. It can be seen that the modified model’s predicted results are highly consistent with the experimental results.

Therefore, the modified Arrhenius-type constitutive model will be rewritten as,

(22) $$\sigma =\frac{1}{\alpha \left(\varepsilon \right)}\sinh^{-1}{\left\{\frac{\dot{\varepsilon }\exp\left[\frac{Q\left(\varepsilon ,\dot{\varepsilon },T\right)}{RT}\right]}{A\left(\varepsilon ,\dot{\varepsilon },T\right)}\right\}}^{\frac{1}{n\left(\varepsilon ,T\right)}}$$

where the model parameters can be expressed as follow,

(23) $$\left\{ \begin{array}{c} \alpha \left(\varepsilon \right)=0.01209-0.07008\varepsilon +0.40198\varepsilon^{2}-1.32864\varepsilon^{3}+2.54498\varepsilon^{4}-2.59908\varepsilon^{5}+1.08967\varepsilon^{6}\\ n\left(\varepsilon ,T\right)=7.66322+14.48418\varepsilon +0.00582T+1.68993\varepsilon^{2}-0.00000446633T^{2}-0.01402\varepsilon T\\ K\left(\varepsilon ,\dot{\varepsilon }\right)=5.99546+3.06765\varepsilon -0.16104\ln\dot{\varepsilon }+2.92598\varepsilon^{2}+0.04702{\left(\ln\dot{\varepsilon }\right)}^{2}-0.48355\varepsilon (\ln\dot{\varepsilon })\\ Q\left(\varepsilon ,\dot{\varepsilon },T\right)=\mathrm{Rn}\left(\varepsilon ,T\right)K\left(\varepsilon ,\dot{\varepsilon }\right)\\ b\left(\varepsilon ,T\right)=-141.54581-2.60296\varepsilon +0.1947T+2.3216\varepsilon^{2}-0.000065T^{2}+0.000453\varepsilon T\\ \mathrm{lnA}\left(\varepsilon ,\dot{\varepsilon },T\right)=b\left(\varepsilon ,T\right)+\frac{Q(\varepsilon ,\dot{\varepsilon },T)}{RT} \end{array}\right.$$

**Figure 17:**
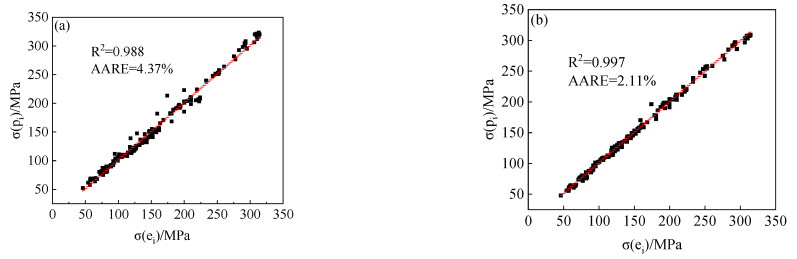
The relative error curves of the prediction results: (**a**) the Arrhenius-type model, (**b**) the modified Arrhenius-type model.

In order to quantificationally analyze the accuracy of the Arrhenius-type model and the modified Arrhenius-type model established in this paper, the relative error curves of the prediction results of these two models and the test results were obtained, as shown in Figure 17. It can be seen from the relative error results that the predicted accuracy of the modified Arrhenius-type model is significantly higher than the Arrhenius-type model. Therefore, the modified Arrhenius-type model, which comprehensively considers the effects of strain, temperature, and strain rate on model parameters, can better predict the flow stress of 34CrNi3Mo steel under high temperature deformation.

## 4. Thermal Deformation Behavior

### 4.1. Evolution of Activation Energy

**Figure 18:**
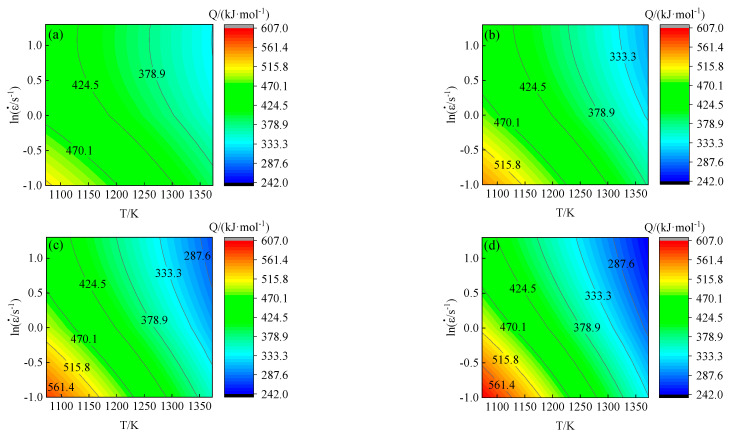
The evolution of activation energy: (**a**) *ε* = 0.15, (**b**) *ε* = 0.3, (**c**) *ε* = 0.45, (**d**) *ε* = 0.6.

The evolution of activation energy Q of 34CrNi3Mo steel at different temperatures and strain rates was calculated by the Arrhenius-type model, as shown in Figure 18. It can be seen from the figure that the evolution of activation energy of 34CrNi3Mo steel varies from 242 kJ·mol^−1^ to −607 kJ·mol^−1^ under different deformation conditions and decreases with the increase of deformation temperature and strain rate. This indicates that the dislocation motion of 34CrNi3Mo steel is promoted by temperature under the high temperature. At the same time, the softening effect of temperature is obviously enhanced with the increase of deformation temperature. This will cause the dislocation density and the activation energy to decrease. Under the condition of high strain rate, the high deformation rate increases the shear stress and is favorable to the dislocation movement, thus reducing the dislocation density.

### 4.2. Processing Map

According to the dynamic material model (DMM), the total energy absorbed by the workpiece during the thermal process can be expressed as,

(24) $$P=\sigma \;\dot{\varepsilon }=G+J=\int_{0}^{\varepsilon ˙}\sigma d\dot{\varepsilon }+\int_{0}^{\sigma }\dot{\varepsilon }d\sigma$$

where *P* represents the external input energy, *G* represents the energy consumed by the plastic deformation, and *J* represents the energy dissipation caused by the transformation of microstructures, and these two energy distribution relationships are affected by the constitutive model. The proportion of the energy parameters *G* and *J* is determined by the strain rate sensitivity index *m*.

(25) $$m=\frac{\partial J}{\partial G}=\frac{\dot{\varepsilon }\partial \sigma }{\sigma \partial \dot{\varepsilon }}={\frac{\partial \left(\mathrm{lg}\sigma \right)}{\partial \left(\mathrm{lg}\dot{\varepsilon }\right)}|}_{\varepsilon ,T}\;$$

When the material meets the satisfied linear power dissipation system, the strain rate sensitivity index *m* = 1. On this condition, the energy dissipation *J* reaches the maximum value *J_max_*.

(26) $$J=G=J_{max}=\frac{P}{2}=\frac{\sigma \dot{\varepsilon }}{2}$$

When *m* > 1, the material tends to form twins or shear bands during deformation.

*η* is the energy dissipation factor and its value is *J*/*J*_max_. The energy dissipation factor is used to represent the ratio of microstructure conversion dissipation energy to ideal linear dissipation energy. According to the definition of the energy dissipation factor *η* and the Murty criterion [26], the power dissipation coefficient can be obtained as follows,

(27) $$\eta =\frac{J}{J_{max}}=\frac{P-G}{J_{max}}=2\left\{1-\frac{1}{\sigma \dot{\varepsilon }}\left[{\left(\frac{\sigma \dot{\varepsilon }}{m+1}\right)}_{\dot{\varepsilon }={\dot{\varepsilon }}_{min}}+\int_{{\dot{\varepsilon }}_{min}}^{\dot{\varepsilon }}\sigma d\dot{\varepsilon }\right]\right\}=\frac{2m}{m+1}$$

When the constitutive model is exponential, the expression of energy dissipation factor *η* can be simplified as,

(28) $$\eta =\frac{2m}{m+1}\;$$

The energy consumed in the thermal deformation process consists of two parts, one part is the energy consumed by the dynamic recovery and recrystallization of the material, and the other part is the energy consumed by the microscopic defects, such as holes, adiabatic shear bands, and wedge cracks. Therefore, the larger the value of the energy dissipation factor, the greater the change in microstructure. However, it is not the higher the energy dissipation rate, the better the thermoforming formability.

The flow instability criterion proposed by Prasad [25] can be used as the instability criterion for 34CrNi3Mo steel during thermal deformation, thus the instability factors $\zeta \left(\dot{\varepsilon }\right)$ can be expressed as,

(29) $$\zeta \left(\dot{\varepsilon }\right)=\frac{\partial \ln\left(\frac{m}{m+1}\right)}{\partial ln\dot{\varepsilon }}+m<0$$

From Equation (29), it can seen that the function of strain rate and deformation temperature. When $\zeta \left(\dot{\varepsilon }\right)$ < 0, the fluidity instability will occur.

**Figure 19:**
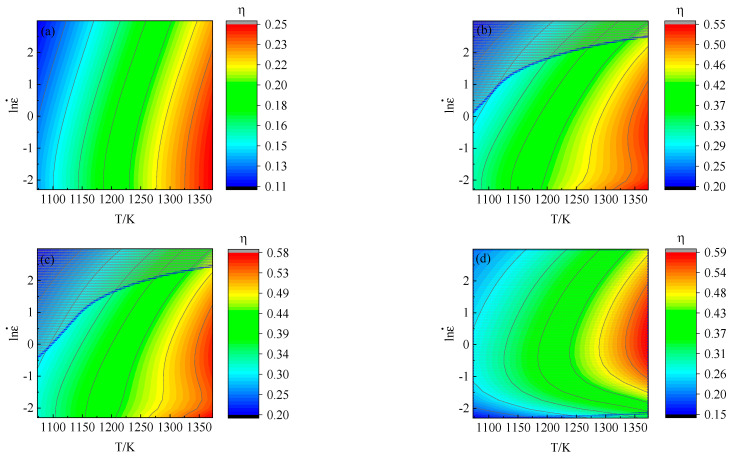
The processing map under the different strain conditions: (**a**) *ε* = 0.15, (**b**) *ε* = 0.3, (**c**) *ε* = 0.45, (**d**) *ε* = 0.6.

Based on the experimental results and Equations (28) and (29), the energy dissipation diagram and plastic instability diagram can be drawn, respectively. Then, the processing map of 34CrNi3Mo steel can be obtained by superposition the energy dissipation diagram and the plastic instability diagram, as shown in Figure 19. In the processing map, the contour line represents the percentage of power dissipation efficiency, and the shaded part represents the plastic instability area.

Comparing the processing map under different strain conditions, the area of the plastic instability zone increases gradually with the increase of strain. When the deformation temperature is constant, the lower the strain rate, the less possibility of plastic instability. Under the same strain rate condition, the power dissipation efficiency increases gradually with the increase of deformation temperature, at the same times, the driving force of microstructure change increases gradually. The comprehensive analysis shows that the optimum deformation temperature and strain rate of 34CrNi3Mo steel should be higher than 1273 K and less than 10 s^−1^.

## 5. Conclusions

(1) The flow stress of 34CrNi3Mo steel in the thermal deformation process is the result of the interaction of work hardening, dynamic recovery, and recrystallization. Under the same deformation rate, with the increase of deformation temperature, the softening effect of dynamic recrystallization and dynamic recovery increases gradually, and the flow stress decreases gradually. However, under the same deformation temperature, with the increase of strain rate, the influence of work hardening on 34CrNi3Mo steel gradually dominates, and the flow stress increases gradually.

(2) The modified Arrhenius model can accurately reflect the influence of temperature, strain rate, and strain on flow stress under thermal deformation conditions, and it can also accurately predict the flow stress of 34CrNi3Mo steel under thermal deformation process.

(3) The evolution of the activation energy indicates that the deformation process of 34CrNi3Mo steel is a thermal activation process, which is affected by temperature, strain rate, and strain. With the increase of deformation temperature and strain rate, the value of deformation activation energy decreases gradually.

(4) According to the dynamic material model, the processing map of 34CrNi3Mo steel was established. At the same deformation temperature, the lower the strain rate, the less prone to plastic instability of 34CrNi3Mo steel. At the same strain rate, with the increase of deformation temperature, the efficiency of power dissipation gradually increases during plastic deformation, and the driving force for microstructure change of 34CrNi3Mo steel gradually increases. The optimal deformation temperature of 34CrNi3Mo steel should be higher than 1273 K and the strain rate should be less than 10 s^−1^.

## Figures and Tables

**Table 1:** The values of *n*_1_, *β,* and *α* under different deformation conditions.

Temp./K	1073	1223	1323	1373	Average
*n*_1_	15.873	10.425	7.7972	6.8264	10.231
*β*	0.0605	0.0654	0.0676	0.0713	0.0662
*α*	0.0038	0.0062	0.0087	0.0104	0.0064

**Table 2:** The values of *n* under different deformation temperatures.

Temp./K	1073	1223	1323	1373	Average
*n*	8.51745	7.75401	6.5601	6.0217	7.2133

**Table 3:** The values of *Q* under different deformation rates.

Strain Rate/s^−1^	0.1	1	10	20	Average
*Q*	443.084	398.4061	357.6501	376.2161	397.4488

**Table 4:** The model parameter values under different strain conditions.

Strain	Q/MPa	n/MPa	LnA	α
0.05	399.1256	7.38668	38.27542	0.00969
0.1	400.6295	7.40179	38.36398	0.008
0.15	402.4933	7.46533	38.45559	0.00724
0.2	402.38308	7.42159	38.43599	0.00684
0.25	402.62062	7.36242	38.53199	0.00659
0.3	397.44876	7.2133	38.09718	0.00647
0.35	391.81027	7.05774	37.64784	0.00638
0.4	386.26765	6.93568	37.16344	0.00634
0.45	381.4456	6.82569	36.74357	0.00634
0.5	378.517	6.78605	36.51825	0.00632
0.55	375.50975	6.72715	36.24138	0.00633
0.6	376.62529	6.77065	36.36216	0.00633

**Table 5:** Polynomial coefficients of material parameters.

**B_0_**	**B_1_**	**B_2_**	**B_3_**	**B_4_**	**B_5_**	**B_6_**
0.01209	−0.07008	0.40198	−1.32864	2.54498	−2.59908	1.08967
**C_0_**	**C_1_**	**C_2_**	**C_3_**	**C_4_**	**C_5_**	**C_6_**
7.64236	−10.40617	139.43461	−761.53963	1911.63147	−2288.30462	1068.61673
**D_0_**	**D_1_**	**D_2_**	**D_3_**	**D_4_**	**D_5_**	**D_6_**
442.8039	−1133.84	11,184.9	−50,811.3	115,013	−128,989	57,596.7
**E_0_**	**E_1_**	**E_2_**	**E_3_**	**E_4_**	**E_5_**	**E_6_**
1.10 × 10^17^	−2.03 × 10^18^	2.21 × 10^19^	−1.06 × 10^20^	2.43 × 10^20^	−2.67 × 10^20^	1.14 × 10^20^

## Data Availability

The data that support the findings of this study are available from the corresponding author, [Xiang-Dong Jia], upon reasonable request.

## References

[1] Luo Y., Guo H., Guo J., Yang W. (2018). Gleeble-Simulated and Semi-Industrial Studies on the Microstructure Evolution of Fe-Co-Cr-Mo-W-V-C Alloy During Hot Deformation. Materials.

[2] Yang X., Li W., Ma J., Hu S., He Y., Li L., Xiao B. (2016). Thermo-Physical Simulation of the Compression Testing for Constitutive Modeling of GH4169 Superalloy During Linear Friction Welding. J. Alloy. Compd..

[3] Haghdadi N., Martin D., Hodgson P. (2016). Physically-based constitutive modelling of hot deformation behavior in a LDX 2101 duplex stainless steel. Mater. Des..

[4] He T., Huo Y., Shi X., Chen S. (2019). Modeling of Carbide Spheroidization Mechanism of 52100 Bearing Steel Under Warm Forming Conditions. Metall. Mater. Trans. A.

[5] Liu L., Wu Y.X., Gong H., Wang K. (2019). Modification of constitutive model and evolution of activation energy on 2219 aluminum alloy during warm deformation process. Trans. Nonferrous Met. Soc. China.

[6] Wang M.H., Yang Y.C., Tu S.L., Wei K. (2019). A modified constitutive model and hot compression instability behavior of Cu-Ag alloy. Trans. Nonferrous Met. Soc. China.

[7] Xiao Y.H., Guo C., Guo X.Y. (2011). Constitutive modeling of hot deformation behavior of H62 brass. Mater. Sci. Eng. A.

[8] Peng W.W., Zeng W.D., Wang Q.J., Yu H. (2013). Comparative study on constitutive relationship of as-cast Ti60 titanium alloy during hot deformation based on Arrhenius-type-type and artificial neural network models. Mater. Des..

[9] Wang L., Liu F., Cheng J.J., Zuo Q., Chen C.F. (2016). Arrhenius-type-Type Constitutive Model for High Temperature Flow Stress in a Nickel-Based Corrosion-Resistant Alloy. J. Mater. Eng. Perform..

[10] Li C.S., He S., Ren J., Han Y. (2021). The Flow Stress Behavior and Constitutive Model of Cr8Mo2SiV Tool Steel during Hot Deformation. Steel Res. Int..

[11] Safari A., Imran M., Weiss S. (2021). A Comparative Study on Modified Johnson–Cook and Arrhenius-type-Type Constitutive Models to Predict the Hot Deformation Behaviour of Molybdenum-Hafnium-Carbide Alloy. J. Mater. Eng. Perform..

[12] Tao Z., Yang H., Li H., Ma J., Gao P. (2016). Constitutive modeling of compression behavior of TC4 tube based on modified Arrhenius-type and artificial neural network models. Rare Met..

[13] Wang J., Yuan X., Jin P., Ma H., Shi B., Zheng H., Chen T., Xia W. (2020). Study on modified Johnson-Cook constitutive material model to predict the dynamic behavior Mg-1Al-4Y alloy. Mater. Res. Express.

[14] Mirzadeh H. (2015). Constitutive modeling and prediction of hot deformation flow stress under dynamic recrystallization conditions. Mech. Mater..

[15] Martins J.M.P., Thuillier S., Andrade-Campos A. (2021). Calibration of a modified Johnson-Cook model using the Virtual Fields Method and a heterogeneous thermo-mechanical tensile test. Int. J. Mech. Sci..

[16] Zhang Y.B., Song Y., Hong X., Wang Z.-G. (2017). A modified Johnson–Cook model for 7N01 aluminum alloy under dynamic condition. J. Cent. South Univ..

[17] Li Z., Wang J., Yang H., Liu J., Ji C. (2020). A Modified Johnson-Cook Constitutive Model for Characterizing the Hardening Behavior of Typical Magnesium Alloys under Tension at Different Strain Rates: Experiment and Simulation. J. Mater. Eng. Perform..

[18] Liu D., Li B., Guo Z., Huang Z. (2021). Finite element analysis on electromagnetic forming of DP780 high-strength steel sheets. Int. J. Adv. Manuf. Technol..

[19] Ding F.J., Jia X.D., Hong T.J., Xu Y.L. (2020). Prediction model on flow stress of 6061 aluminum alloy sheet based on GA-BP and PSO-BP neural networks. Rare Met. Mater. Eng..

[20] Wang M.H., Wang G.T., Wang R. (2016). Flow stress behavior and constitutive modeling of 20MnNiMo low carbon alloy. J. Cent. South Univ..

[21] Ashtiani H.R., Shayanpoor A.A. (2021). Hot Deformation Characterization of Pure Aluminum Using artificial neural network (ANN) and Processing Map Considering Initial Grain Size. Met. Mater. Int..

[22] Quan G.Z., Zou Z.Y., Tong W., Liu B., Li J.-C. (2016). Modeling the Hot Deformation Behaviors of As-Extruded 7075 Aluminum Alloy by an Artificial Neural Network with Back-Propagation Algorithm. High Temp. Mater. Process..

[23] Xi S.P., Gao X.L., Liu W., Lu Y.-L., Fu G.-Q., Tao H.-C., Zang Y.-C. (2021). Hot deformation behavior and processing map of low-alloy offshore steel. J. Iron Steel Res. Int..

[24] Zhao H., Qi J., Su R., Zhang H., Chen H., Bai L., Wang C. (2020). Hot deformation behaviour of 40CrNi steel and evaluation of different processing map construction methods. J. Mater. Res. Technol..

[25] Prasad Y.V.R.K., Gegel H.L., Doraivelu S.M., Malas J.C., Morgan J.T., Lark K.A., Barker D.R. (1984). Modeling of dynamic material behavior in hot deformation: Forging of Ti-6242. Metall. Trans. A.

[26] Murty S.V.S.N., Rao B.N. (1998). On the development of instability criteria during hotworking with reference to IN 718. Mater. Sci. Eng. A.

[27] Jia X.D., Wang Y.N., Zhou Y., Cao M.Y. (2021). The Study on Forming Property at High Temperature and Processing Map of 2219 Aluminum Alloy. Met.-Open Access Metall. J..

[28] Chen X., Liao Q., Niu Y., Jia Y., Le Q., Ning S., Hu C., Hu K., Yu F. (2019). Comparison study of hot deformation behavior and processing map of AZ80 magnesium alloy casted with and without ultrasonic vibration. J. Alloy. Compd..

[29] Yuan C.H., Liu B., Liu Y.X., Liu Y. (2020). Processing map and hot deformation behavior of Ta-particle reinforced TiAl composite. Trans. Nonferrous Met. Soc. China.

[30] Gao P., Chen L., Luo R., Peng C.-T., Sheng D., Liu T., Cheng X. (2021). Investigation of Hot Working Performance and Microstructure Evolution of GH1059 Superalloy Based on Processing Map. Trans. Indian Inst. Met..

[31] Chegini M., Aboutalebi M.R., Seyedein S.H., Ebrahimi G.R., Jahazi M. (2020). Study on hot deformation behavior of AISI 414 martensitic stainless steel using 3D processing map—ScienceDirect. J. Manuf. Process..

